# COVID-19: What paramedics need to know!

**DOI:** 10.1017/cem.2020.367

**Published:** 2020-04-15

**Authors:** Jason E. Buick, Sheldon Cheskes, Michael Feldman, P. Richard Verbeek, Morgan Hillier, Yuen Chin Leong, Ian R. Drennan

**Affiliations:** * Institute of Health Policy, Management and Evaluation, University of Toronto, Toronto, ON; † Sunnybrook Center for Prehospital Medicine, Sunnybrook Health Sciences Center, Toronto, ON; ‡ Division of Emergency Medicine, Department of Family and Community Medicine, University of Toronto, Toronto, ON; § Division of Emergency Medicine, Department of Medicine, University of Toronto, Toronto, ON; ¶ Faculty of Medicine, University of Toronto, Toronto, ON; # Institute of Medical Science, University of Toronto, Toronto, ON

**Keywords:** Emergency medical services, paramedic, prehospital, SARS-CoV-2

## INTRODUCTION

Pandemics are a rare and stressful time for health care providers with overwhelming caseloads, rapidly evolving information, and competing priorities of self-protection, while maintaining a high level of patient care. The goal of this article is to provide paramedics with the necessary information to support their clinical practice during the COVID-19 pandemic.

Two previous coronaviruses caused international responses; the severe acute respiratory syndrome (SARS-CoV-1) in 2003, and the Middle East respiratory syndrome (MERS-CoV) in 2012.^1^ The current coronavirus, SARS-CoV-2, has become a public health emergency and global pandemic. While the terms are often used interchangeably, SARS-CoV-2 refers to the virus, and COVID-19 refers to the illness. For simplicity, we will use the term COVID-19 for both.

## TRANSMISSION

COVID-19 is highly contagious, resulting in exponential growth in the number of patients. The reproduction number (R_0_), the number of individuals who can be infected by one patient, is between 2.2 and 3.6,^2,3^ considerably higher than seasonal influenza (R_0_ 0.9–2.1).^1^

Initially, it was thought it was only transmitted through droplets produced when an infected person coughed or sneezed.^1,2^ COVID-19 is believed to be transmitted through droplets (> 5–10 μm diameter) entering the body by means of the airway and mucous membranes, not though airborne particles (< 5 μm diameter).^1,2^ However, there is a growing body of evidence that community spread is occurring, even from asymptomatic individuals.^1,4,5^

## PRESENTATION

The incubation period for COVID-19 is between 1 and 14 days (average, 5 days).^6^ The majority of adults experience mild symptoms, with only 15% experiencing severe symptoms and 5% becoming critically ill.^4,7^ Elderly patients and those with comorbid conditions such as hypertension, diabetes, cardiovascular disease, and lung disease, are more likely to experience severe cases and death.^4^ Fortunately, as in adults, the majority of pediatric patients present with mild or moderate symptoms, with only 6% classified as severe or critical.^8^

The symptoms of COVID-19 are diverse for both adults and children, and patients do not always present with classic respiratory symptoms (Table 1).^9^ One study found that up to 55% of adults were afebrile upon presentation to hospital.^7^

**Table 1.** Symptoms of COVID-19 infection^9^

Adult patients	Pediatric patients
**Common symptoms**	**Common symptoms**
Cough	Cough
Fever	Fever
Shortness of breath	Throat swelling
Muscle and joint pain	
Fatigue	
**Less frequent symptoms**	**Less frequent symptoms**
Nausea, vomiting, diarrhea	Nausea, vomiting, diarrhea
Headache	Fatigue
Sore throat	Runny nose

Given the wide range of nonspecific symptoms, paramedics should have a high index of suspicion and exercise caution when a patient presents with a history suggestive of possible COVID-19, using contact/droplet or airborne precautions as required.

## PREVENTION

All patients should be screened for potential symptoms before patient contact starting with the dispatch center. Given the extent of community transmission, patients no longer need a travel history to be considered for having COVID-19.^5^ First responders should remain diligent even after prescreening by dispatchers. Personal protective equipment (PPE) should be used based on the risk of exposure (eg.,. type of activity) and the transmission dynamics of the pathogen (eg.,. contact, droplet, aerosol).^10^

When no interventions are performed, paramedics providing care or who are in close proximity to the patient should wear a surgical type mask, gown, gloves, and eye protection, either goggles or a face shield. Safety glasses are not sufficient for eye protection. For aerosol generating medical procedures (AGMPs), paramedics should wear a N95 mask, eye protection, gloves, and fluid resistant gowns. PPE should be removed while driving the ambulance as long as the driver compartment is separate from the patient. This is important to limit contamination to the front of the ambulance. If there is no separation, the driving paramedic should wear a mask. Additionally, a mask should be provided to the patient as tolerated.^10,11^

Proper use of PPE and frequent hand sanitizing/washing is essential to prevent disease transmission in the health care setting.^1^ In addition, surfaces should be disinfected frequently as the virus remains viable for extended periods of time on different materials, such as copper (4 hours), cardboard (24 hours), stainless steel (48 hours), and plastics (72 hours).^12^

## CLINICAL CONSIDERATIONS

The majority of patients with COVID-19 will experience mild symptoms, and do not require emergency care. These patients should be isolated at home to prevent transmission. For patients who require further treatment and/or transport to hospital, paramedics, must wear appropriate PPE before patient contact or initiating care. Initially, only one paramedic should make patient contact, and perform an initial assessment to determine if additional providers are required. Unnecessary personnel should be removed from the scene to limit exposure, and should not accompany paramedics during transport to the hospital.

### Aerosol Generating Medical Procedures

Airborne transmission is possible when AGMPs are performed. AGMPs include advanced airway insertion, bag-valve-mask (BVM) ventilations, chest compressions, open airway suctioning and tracheostomy care, nebulized treatments, and noninvasive positive pressure ventilation. These procedures should be avoided, unless absolutely necessary.^9,13^ Paramedics should consider alternatives, such as a metered-dose inhaler or parenteral administration of medications (e.g., intramuscular epinephrine), or withholding care in less severe cases.

### Cardiac arrest

When providing ventilations by means of BVM, paramedics should use a two-handed approach (one person holding the mask and another squeezing the bag) to ensure a good seal is being maintained.^9^ In general, two-handed BVM should not lead to exposure of additional paramedics.

Early placement of an advanced airway should be considered. The risk of exposure during intubation may be minimized by using the most experienced and skilled paramedic, video laryngoscopy, a bougie, and pausing chest compressions. This will provide more protection to responders as postintubation ventilation (with appropriate filter and airway cuff pressure) can limit exposure to secretions.^13^

Supraglottic airways (SGAs) may offer an alternative to endotracheal intubation, which may limit exposure during placement. It is not known if SGAs reduce exposure or provide the same level of protection as intubation, but these devices should be considered when a good mask seal with BVM is difficult to achieve (e.g., transportation).

**Figure 1.**
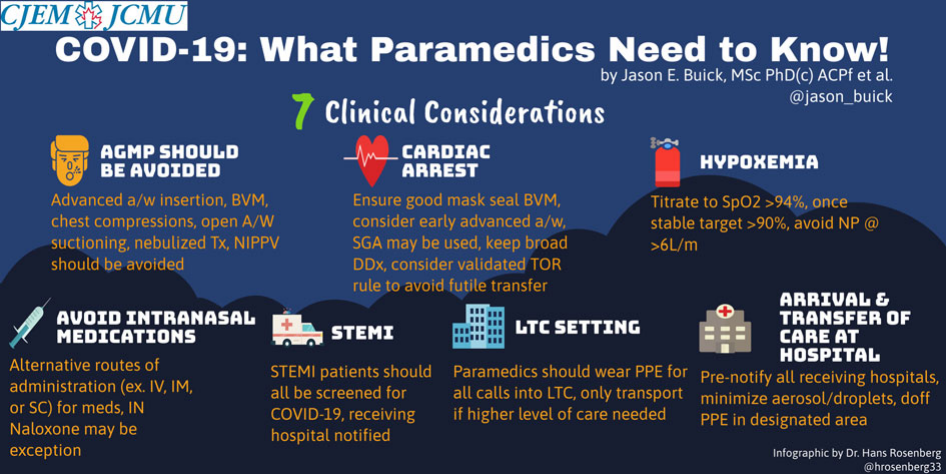
What Paramedics Need to Know infographic.

There is no evidence on outcomes or specific interventions for out-of-hospital cardiac arrests related to COVID-19. Even though patients may have COVID-19, it is important to consider other pathologies of cardiac arrest. Keep a broad differential diagnosis! Paramedics should initiate resuscitation as per standard practice, and use previously validated termination of resuscitation (TOR) rules to limit transportation of nonviable patients. Application of a TOR rule will limit the time of exposure and avoid transfer of futile patients to hospital. In the event of a prolonged surge event that limits intensive care unit (ICU) bed availability, anticipate changes in your protocols that may limit the conditions under which you initiate resuscitation.

### Hypoxemia

Paramedics should consider slowly titrating supplemental oxygen flow rates to achieve an oxygen saturation of >94%. Then once stable, target an SpO2 of 90%.^9,13^ Oxygen flow rates >5–6 L/min are considered aerosol generating and the patient should be switched to a high concentration/low flow mask with an exhalation filter.

### Intranasal medications

Paramedics should use alternative routes of administration (eg.,. intravenous, intramuscular, or subcutaneous) for medication administration. For opioid emergencies, if other routes are not accessible, intranasal naloxone should be prioritized over BVM ventilations. If administered appropriately, the risk for transmission is low, as it produces large droplets which fall into the nasopharynx rather than forming aerosol suspension in the air.

### ST-elevation myocardial infarction

All ST-elevation myocardial infarction (STEMI) patients should be screened for COVID-19. Patients should be transported to the designated percutaneous coronary intervention (PCI) center regardless of COVID-19 status, pending discussion with the interventional cardiologist. The receiving center should be notified early for all positive patients to ensure staff are adequately prepared. Systems should attempt to minimize “false positive” STEMI activations. Patients may be redirected to the emergency department first, as COVID-19 has been associated with myocarditis, which may mimic STEMI on electrocardiogram. Finally, should the PCI lab and medical system become overwhelmed, consideration of thrombolytic therapy may occur in some areas.^14^

### Long-term care setting

Paramedics should wear PPE for all calls into long-term care (LTC) settings regardless of the patient's complaint. Patients in LTC settings can have atypical symptoms of COVID-19 infection or be asymptomatic.^15^ It is important to consider that staff can also be contagious.

Patients should only be transported to hospital when there is a need for a higher level of care than what the LTC setting can provide, not for the purpose of COVID-19 testing. Facilities should contact their local public health unit for guidance or consider using community paramedics to perform testing.^16^

Patients with mild symptoms should not be transported to hospital. However, this can be problematic if patients require isolation or a negative pressure room. If unsure, paramedics should contact online medical control for advice. Whether or not to transport more severely ill patients depends on local ICU capacity and COVID-19 disease activity. These decisions should be discussed at a local level. Transport and treatment decisions should be patient-centered and focused around goals of care. Ensure you have the most up-to-date version of the patients’ advanced directives, as their wishes might have changed with the pandemic.^17^ Paramedics should consider the patient's and/or substitute decision-makers request not to transport the patient to hospital.

### Arrival and transfer of care at hospital

Paramedics should prenotify all receiving hospitals if a patient is suspected of having COVID-19. Resuscitation and other treatment should be continued in the ambulance until directed by receiving staff. Paramedics must be mindful when passing other stretcher patients, colleagues, and other health care providers who are not wearing PPE. Only providers with appropriate PPE should maneuver the stretcher.

While moving a patient between the ambulance and the resuscitation room, steps must be taken to minimize or eliminate aerosol and respiratory droplets. The patient can continue to receive supplemental oxygen and chest compressions, but manual ventilations should be withheld unless the patient has been intubated.

After the call, paramedics should remove their PPE in a designated area under observation of a trained observer. This ensures that proper procedures are being followed to limit any cross-contamination.

## CONCLUSIONS

The COVID-19 pandemic is a rapidly changing global health crisis. The unique challenges of the prehospital setting are magnified in times like this, and we cannot automatically extrapolate hospital care to the unpredictable prehospital environment. Given the rapidly evolving state of affairs related to COVID-19, and the difficulty paramedics have gaining access to up-to-date information, this article provides guidance to paramedics on the best approach to COVID-19 patients in the prehospital setting.

## DISCLAIMER

The views and opinions expressed in this commentary are those of the authors and do not necessarily reflect their organizations, the Canadian Journal of Emergency Medicine, or the Canadian Association of Emergency Physicians. There are variations in protocols, and paramedics should follow their own services, local, national and international policies, procedures, recommendations, and guidelines.
